# Simultaneous inhibition of multiple oncogenic miRNAs by a multi-potent microRNA sponge

**DOI:** 10.18632/oncotarget.4827

**Published:** 2015-07-30

**Authors:** Jaeyun Jung, Chanjoo Yeom, Yeon-Sook Choi, Sinae Kim, EunJi Lee, Min Ji Park, Sang Wook Kang, Sung Bae Kim, Suhwan Chang

**Affiliations:** ^1^ Department of Biomedical Sciences, University of Ulsan School of Medicine, Seoul 138-736, Korea; ^2^ Asan Medical Center, Seoul 138-736, Korea

**Keywords:** oncogenic microRNA, miRNA inhibitor, miRNA sponge, breast cancer, pancreatic cancer

## Abstract

The roles of oncogenic miRNAs are widely recognized in many cancers. Inhibition of single miRNA using antagomiR can efficiently knock-down a specific miRNA. However, the effect is transient and often results in subtle phenotype, as there are other miRNAs contribute to tumorigenesis. Here we report a multi-potent miRNA sponge inhibiting multiple miRNAs simultaneously. As a model system, we targeted miR-21, miR-155 and miR-221/222, known as oncogenic miRNAs in multiple tumors including breast and pancreatic cancers. To achieve efficient knockdown, we generated perfect and bulged-matched miRNA binding sites (MBS) and introduced multiple copies of MBS, ranging from one to five, in the multi-potent miRNA sponge. Luciferase reporter assay showed the multi-potent miRNA sponge efficiently inhibited 4 miRNAs in breast and pancreatic cancer cells. Furthermore, a stable and inducible version of the multi-potent miRNA sponge cell line showed the miRNA sponge efficiently reduces the level of 4 target miRNAs and increase target protein level of these oncogenic miRNAs. Finally, we showed the miRNA sponge sensitize cells to cancer drug and attenuate cell migratory activity. Altogether, our study demonstrates the multi-potent miRNA sponge is a useful tool to examine the functional impact of simultaneous inhibition of multiple miRNAs and proposes a therapeutic potential.

## INTRODUCTION

miRNAs are small endogenous RNAs that can inhibit protein expressions of target mRNAs, by interacting mainly to its 3′UTR and thus degrade mRNAs or inhibit translation [1, 2]. Increasing number of findings demonstrated miRNAs are one of the major regulators in gene expression [3–5], and therefore its regulation became a great interest scientifically as well as in translational medicine. So far, there are number of miRNA-regulating agents being tested in clinical/preclinical trials. These include miR-122 inhibitor for HCV driven hepatitis [6, 7] and anti-miR-34a for cardiovascular diseases [8].

Another miRNA regulating agents is microRNA sponge that is initially introduced on 2007 [9]. Unlike the antagomiR that is a complementary synthetic RNA to the target miRNAs [10, 11], miRNA sponge is a DNA construct that produces artificially designed miRNA binding sites on the 3′UTR region of a non-toxic gene such as GFP or luciferase. The expression of artificial RNA with specific (and usually multiple) miRNA binding sites can absorb endogenous miRNA (thus the name comes in), essentially depleting the target miRNA in cells. Although antagomiR has advantages including ease of synthesis and diverse chemical modification to improve its stability [12, 13], miRNA sponge can achieve stable inhibition as well as inducible / tissue specific inhibition of target miRNAs *in vitro/vivo* [14, 15]. Moreover, inserting a number of different miRNA binding sites generates a miRNA sponge that can simultaneously, functionally inhibit multiple miRNAs [16]. Indeed, a report showed the inhibition of three miRNAs using miRNA sponge [17]. Moreover, recent papers have shown the inhibition of multiple oncogenic miRNAs by miRNA sponges in Ewing sarcoma (targeting miR-106a∼363 cluster) [18] or breast cancer (targeting miR-183/-96/-182 cluster) [19]. Because there are numerous miRNAs known to implicate in cancer [20, 21], these results are valuable in respect that they inhibited a number of miRNAs simultaneously. However, three different miRNAs inhibited in their work were a polycistronic miRNA cluster, leaving a room for a multi-potent miRNA sponge inhibiting several independent miRNAs. In this report, we generated a miRNA sponge inhibiting 4 miRNAs, which are not in a miRNA cluster. Because several miRNAs seems to have common roles in multiple types of cancer, we aimed to examine the effect of “driver miRNAs” inhibition [22, 23]. We selected 4 driver miRNAs to inhibit, based on the previous findings. The first one, miR-155, is implicated in many physiological processes, including differentiation and activation of various immune cells such as T cell, B cell and dendritic cells [24, 25]. It is also a typical oncomiR in lymphoma as well as breast, pancreatic, lung and colon cancers [26–28]. Secondly, miR-21 is known to play a role in heart development and the increased level of miR-21 was detected in heart failing conditions [29, 30]. In cancer, it is one of the well-known oncogenic miRNA inhibiting multiple tumor suppressors including PTEN, MSH2 and JAG1 [31–33]. miR-221 and miR-222 are paralog, identical in the seed sequence and are located in the same genome locus by 727bp apart [34]. It is implicated in angiogenesis, proliferation and cell migration. They are over-expressed in prostate, lung, thyroid and pancreatic carcinoma [34, 35].

We report here a multi-potent miRNA sponge that simultaneously inhibits these 4 well-known oncomiR. The data presented here demonstrate the miRNA sponge is a useful tool to inhibit these miRNAs simultaneously and suggest a potential to use such tool as a therapeutic agent.

## RESULTS

### Design and construction of the multi-potent miRNA sponge

To determine target miRNAs, we reviewed previous reports regarding miRNA expression and functional analysis on breast and pancreatic cancer [27, 28, 31, 35]. We aimed to select miRNAs that showed up-regulated expressions with oncogenic functions in both cancers. As a result, we selected miR-155, miR-21, miR-221/222 and the mature sequences are shown in supplementary table 2.

**Figure 1:**
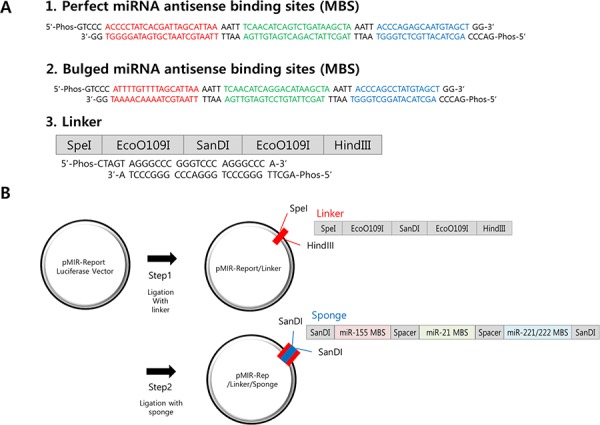
Construction of the multi-potent miRNA sponge **A.** The sequence of perfectly matched (1) or Bulged (2) sponge monomer for miR-155 (red), miR-21 (green) and miR-221/222 (blue). There is a spacer (AATT) added after each miRNA-binding site. A SanDI linker was added at each end the sponge monomer for directional cloning. Linker (3) was designed to introduce SanDI site into the reporter plasmid (pMIR-Report). **B.** Two-steps for multimeric miRNA sponge construction. First the linker was inserted through SpeI/HindIII sites and secondly, sponge monomer was directionally ligated into the SanDI site. By changing the molar ratio of the vector to insert, multimerized sponge vectors are generated and confirmed by sequencing.

In order to inhibit these miRNAs simultaneously, we designed oligonucleotide containing different miRNA binding sites (MBS) tandemly, with a short spacer (AATT, in Figure 1A). The unit was referred as “monomer”. We reasoned that the spacer would reduce non-specific binding of miRNAs and generate enough space so several miRNAs can bind to MBS stably without overlapping on each other [16]. The multi-potent miRNA sponge vector was generated by introducing SanDI restriction enzyme site (GGGTCCC) at the end of the monomer, thereby it can be directionally cloned. For the efficient binding of each miRNA, the MBS sequence was designed to be reverse complementary (Perfect) for the matured miRNA sequences. For the MBS of miR-221 and 222, we introduced a common nucleotide sequence (agcuacauugcucugggu) as they are identical in their seed sequence and only 4 bases are mismatched in full miRNA sequence. Combining with the miR-155 and miR-21 MBS, the sponge is considered to have binding sites for 4 miRNAs (miR-155, 21, 221, 222) in total.

On the other hand, we also designed MBS with 2–3 bases of mismatch (Bulged), as some of reports suggested it can mimic physiological miRNA-target interaction [36], thereby have better potential as a sponge. We created bulged form by deletion of one nucleotide and changing the remaining three nucleotides in such a way that chance of base pairing (including G-U wobbling) is minimal [16]. In this method, MBS is commonly the antisense sequence of miRNA with a central mismatch at position 9–12 of the miRNA sequence. In our sponge, for the sequence of miR-155, it was designed to have seed sequence of miR-155 and G-U wobbling sequence between 10^th^ and 12^th^ nucleotide. Also, base paring of the last part of the sponge and miR-155 is minimal, which accords with the condition of bulged sponge. In the case of miR-221 and 222, bulged form has changed sequence at position 9–12^th^ nucleotide of miRNA and one nucleotide was deleted. We used pMIR-Report vector as a backbone (Figure 1B) because the inhibition of gene expression would be easily measured by luciferase assay. Combining these ideas, we were able to obtain miRNA sponges with one to three copies of perfect matching MBS or one to five copies of bulged MBS.

### Effective inhibition of luciferase activity by the 3′ insertion of prefect or bulged MBS

In order to test the luciferase-based miRNA sponge with different numbers of MBS can inhibit miRNAs efficiently, we transfected these sponges into MDA-MB-436 breast cancer cell first and measured luciferase activity. We used MDA-MB-436 cells based on the real-time PCR quantification results for the 4 target miRNAs in 5 cell lines, which revealed this cell line is relatively high for the target miRNAs (Supplementary Figure 1).

**Figure 2:**
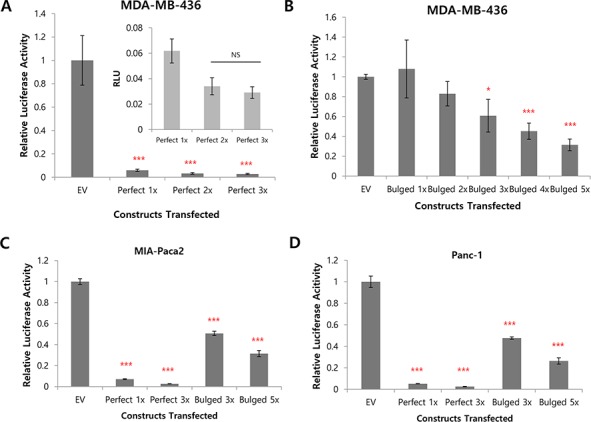

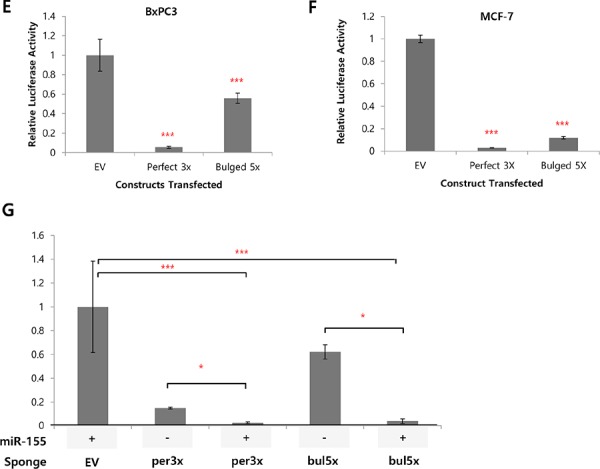
Efficient knockdown of luciferase activity by the 3′UTR insertion of MBS **A, B.** luciferase activity of control (EV, empty vector) or perfectly matched (A) or bulged (B) sponge in MDA-MB 436 cells, with different copy numbers of sponge monomer inserted. For perfectly matched sponge, 1 to 3 copies were tested (A) whereas 1 to 5 copies were tested for bulged sponge (B). The inset graph in (A) is for the perfect 1X∼perfect 3X enlarged. NS: Not Significant. **C, D.** Luciferase activity for the same perfect (1 and 3 copies) or bulged (3 and 5 copies) sponge in pancreatic cancer cells MIA-PaCa2 (C), Panc-1 (D). **E, F.** Efficient knockdown of luciferase by perfect (3X) or bulged (5X) sponge in BxPC3 (E) and MCF7 (F) cells. **G.** MCF7 was transfected with sponge alone or with miR-155 over-expression vector and luciferase activity was measured. Note a significant decrease of luciferase activity by the over-expressed miR-155 for both of the perfect or bulged sponges. *p < 0.05, **p < 0.01, ***p < 0.001

Because miRNA inhibits protein expression in general, reduction of the luciferase activity in the sponge-transfected cells compared to the EV (empty vector; no MBS inserted) reflects the degree of miRNA inhibition through the MBS-miRNA binding. Compared to the luciferase vector without MBS sites (EV), we indeed observed dramatic decrease of the luciferase activity when the perfect matching MBS are present (Figure 2A). This data suggest the MBS sites are actively absorbing miRNAs (so the name is sponge), resulting in the reduced expression of the luciferase. A single copy of the perfect MBS was enough to be efficiently knockdown the luciferase activity, even though increased copy of MBS (2X or 3X) slightly reduced the luciferase activity further (Figure 2A, inset). In contrast, we observed a copy number dependent, gradual decrease of the luciferase activity for bulged sponge expression (Figure 2B, see discussion). We verified these results in four other cancer cell lines including MIA-Paca2, Panc-1, BxPC3 and MCF-7 (Figure 2C∼2F). All of the four cell lines showed efficient reduction of the luciferase activity by the perfect or bulged MBS. Additionally, when we over-expressed miR-155 in MCF-7 cells (with low endogenous miR-155, supplementary Figure 1A), we observed further reduction of the luciferase activity (Figure 2G). This data suggest the miRNA sponge can absorb abnormally induced miRNAs as well.

### Construction of RFP-based inducible sponge and stable cell line

**Figure 3:**
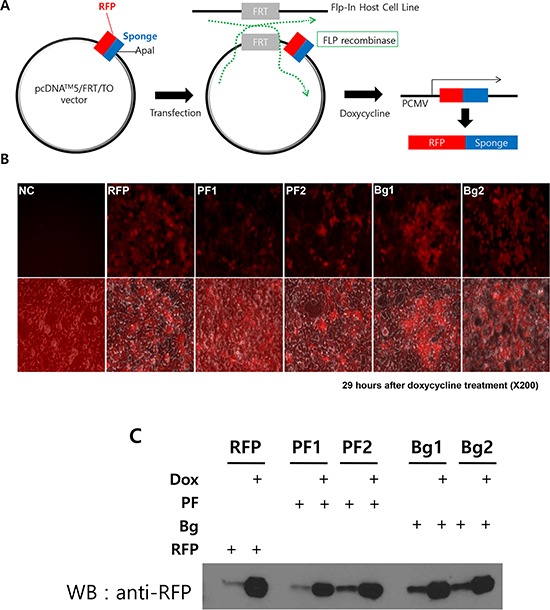
Generation of inducible RFP-miRNA sponge system in Flp-In T-Rex 293 cells **A.** Diagram showing how the inducible miRNA sponge cell line was generated. RFP-sponge fusion DNA was introduced into the inducible expression vector. Then the construct was transfected into Flp-In T-REx293 cell to induce targeted integration, which is selected by hygromycin. Doxorubicin treatment induces RFP sponge fusion transcript. **B.** Fluorescence imaging of the RFP-sponge induced by doxycycline. Flp-In TREx293 cells with inducible RFP-sponge vector was treated with doxycycline for 29 hours and the expression of RFP was checked under fluorescence microscope. The bottom row of picture shows fluorescent images, which were merged with images taken in the phase contrast. (NC: Negative Control; RFP: RFP without MBS; PF1, PF2: two independent cells with perfectly matched sponge unit; Bg1, Bg2: two independent cells with bulged sponge unit). **C.** Western blot analysis of induced RFP protein. Total extracts from the same cells in B were analyzed. Note that uninduced cells expressed marginal level of RFP.

Based on the results above, we next examined the effect of the sponge on the expression and functionality of the individual miRNA. To do this experiment, we developed inducible, RFP (Red Fluorescence Protein) based miRNA sponge (Figure 3A). Although the transient transfection of the miRNA sponge vector can produce high level of sponge, the functional analysis is dependent on transfection efficiency. In addition, using fluorescence protein gene as a sponge backbone makes expression analysis easier (by microscopy) and enables sorting of the sponge positive cells using FACS. Therefore, we introduced RFP gene instead of luciferase at the upstream of sponge sequences. Also, we selected an inducible promoter (Tetracycline inducible in pcDNA5/FRT/TO/RFP) to control the expression of the sponge in cancer cell. We introduced the same miRNA sponge units (both perfect and bulged) into the inducible vector to generate inducible RFP-miRNA sponges. The constructs were then introduced into the Flp-In TREx 293 cells. This cell line contains a FLP locus where the sponge vector can be integrated by the FLP recombinase, resulting in a cell line with homogenous, inducible miRNA sponge expression (Figure 3A). We generated two independent clonal cells for each type of the sponge, marked as PF1/PF2 or Bg1/Bg2 respectively. For each cell line, robust induction of RFP by doxycycline (tetracycline analog) was detected by fluorescence microscopy as well as western blot analysis shown in Figure 3B and 3C.

### Molecular analysis of the inducible sponge cells reveals the multi-potent miRNA sponge inhibits 4 target miRNAs functionally

**Figure 4:**
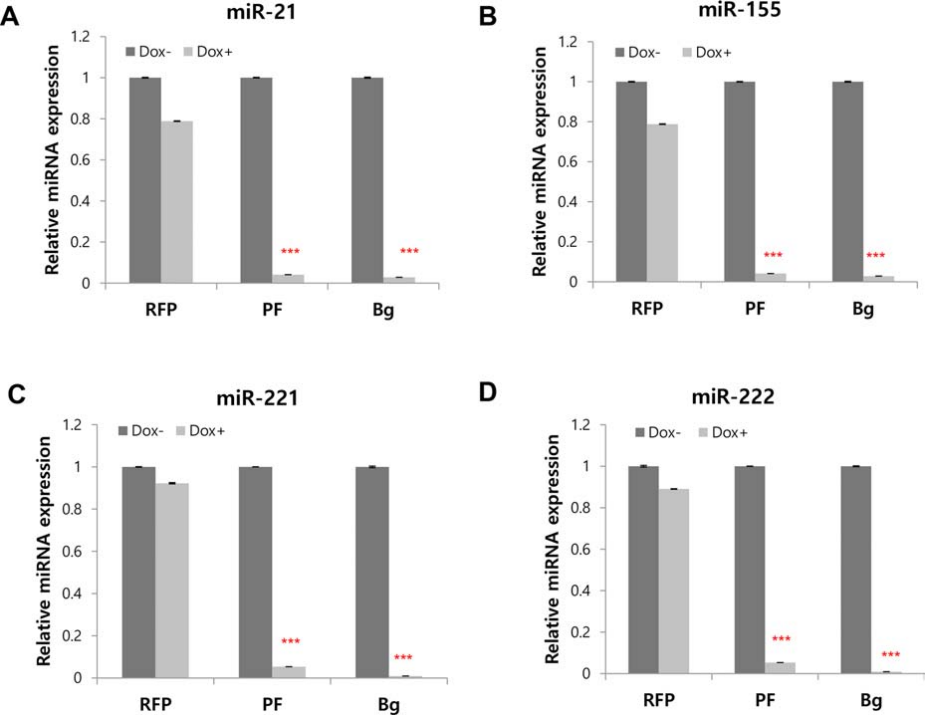

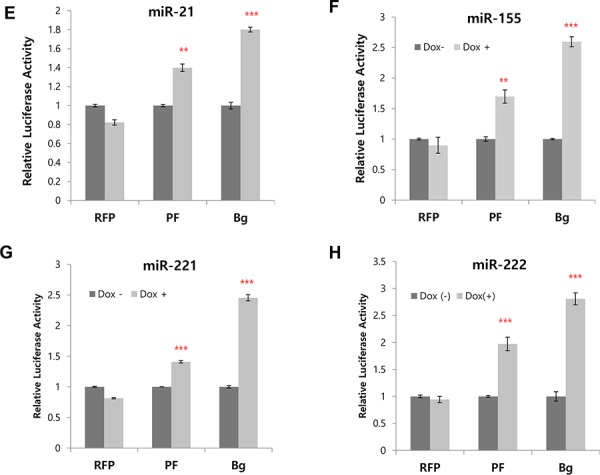

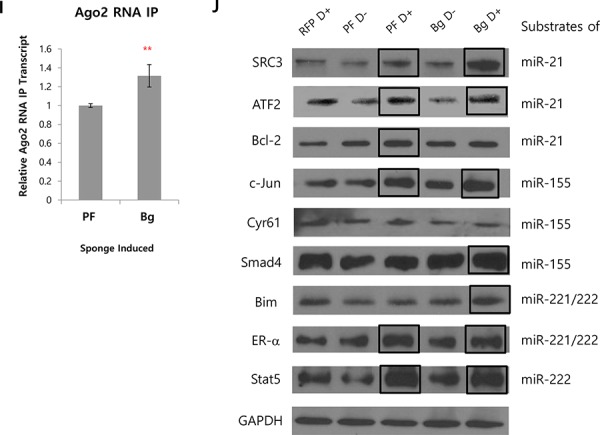
Simultaneous inhibition of 4 miRNAs and concomitant protein level changes by multi-potent miRNA sponge **A–D.** Real time PCR analysis of 4 target miRNAs after induction of RFP based miRNA sponge. The level of miR-155, miR-21, miR-221 and miR-222 were measured after 24 hrs of induction (RFP : without MBS; PF: perfectly matched sponge; Bg: Bulged sponge). **E–H.** Dual luciferase assay was performed after the transfection of reporter plasmid for each targeted miRNA. The primer sequences for reporter plasmids were shown in supplementary Table 1. **I.** Real time PCR analysis of perfect and bulged sponge transcripts after immunoprecipitation with Ago2 antibody, representing the amount of each sponge loaded in the Ago2-containing microRNA:mRNA complex. **J.** Western blot analysis of target proteins for 4 miRNAs inhibited by the miRNA sponge. 2∼3 known targets for each of the 4 target miRNAs were selected and the protein levels were analyzed in Flp-In TREx 293 cells after the induction of the miRNA sponge. (RFP: control without sponge unit; D: Doxycycline; PF: perfectly matched sponge; Bg: Bulged sponge). Bold boxes indicate targets that showed increased protein level after induction of the sponge. *p < 0.05, **p < 0.01, ***p < 0.001

Using the inducible sponge cell line, we questioned whether the induced miRNA sponge could inhibit all of the 4-targeted miRNAs simultaneously. To this goal, the level of each target miRNA was measured by real-time PCR after the induction of miRNA sponge. As shown in Figure 4A–4D, we observed dramatic reduction of all of the four miRNAs upon the expression of Perfect (PF) or Bulged (Bg) sponges.

To confirm the inhibitory effect of the miRNA sponge, we made reporter plasmids having MBS for each of the target miRNA. Relative luciferase activity was measured after each plasmid was transfected in Flp-In TRex 293 cell where sponge was induced by doxycycline. We found increased reporter activity for all target miRNA reporters, showing the miRNA sponge functionally inhibits target miRNAs (Figure 4E–4H).

As we observed stronger inhibitory effect of bulged miRNA sponge than perfect in the inducible cells, we compared the amount of perfect and bulged sponge transcript loading on the miRISC complex. After inducing the sponge by treatment of doxycycline in Flp-In TRex 293 cells, RNA immunoprecipitation with Ago2 antibody and real-time PCR were performed. The result shows slight but significantly more bulged sponge transcript in Ago2 IP product, suggesting that bulged sponge was more efficiently loaded in miRISC complex than perfect sponge (Figure 4I).

Based on this result, we next tested the expression changes of proteins that are known to be regulated by 4 target miRNAs, after the induction of the miRNA sponge. We selected 9 target proteins by checking previous literatures and validated miRNA target database (miRTarBase; http://mirtarbase.mbc.nctu.edu.tw). After 48 hrs of doxycycline induction, the cells were harvested and analyzed by western blot with antibodies recognizing each of the protein. As shown in Figure 4J, we could observe increased protein levels for some, not all (see discussion), of the known targets after the induction of miRNA sponge. That includes Bim, ER-alpha and Stat5 for miR-221/222; SRC3, ATF2 and Bcl-2 for miR-21; C-Jun and Smad4 for miR-155. These results indicate that expression of the multi-potent miRNA sponge up-regulates target protein expressions by successfully inhibiting the 4 target miRNAs.

### Prediction of and validation of the target specificity of the multi-potent miRNA sponge

As we introduced spacers and tandem repeats of MBS in the miRNA sponge, we questioned if there is any nonspecific miRNA interaction on the sponge constructs. To answer for this, we analyzed MBS sequences in miRNA prediction program (PITA, URL: http://genie.weizmann.ac.il/pubs/mir07/mir07_prediction.html).

The program generates a list of miRNA candidates in the order of the affinity on the input sequence, determined by free energy changes by the miRNA-MBS interaction. As shown in Tables 1 and 2, our miRNA sponge sequence was predicted to primarily target by the four miRNAs we aimed to inhibit. However, there are other miRNAs following these four with possible binding. Therefore, we decided to analyze four of these “next-ranked” non-specific miRNAs. The results are shown in supplementary figure 2, showing that the level of the change for the four non-specific miRNAs (miR-760, miR-361-5p, miR-196b and miR-485-5p in the Tables 1 and 2) is marginal. For further assessing specificity of the miRNA sponge, we constructed individual reporter plasmids for these “next-ranked” non-specific miRNAs. Luciferase reporter assay showed no difference between the control and the sponge-induced group, confirming that miRNA sponge did not affect activities of these miRNAs (Supplementary Figure 3).

**Table 1:** List of miRNAs predicted to bind to the perfect miRNA sponge (MBS)

microRNA	Position^1^	Seed^2^	dGduplex^3^	dGopen^4^	ddG^5^
hsa-miR-155	20	8:0:0	−38.5	−3.22	−35.27
hsa-miR-21	46	8:0:0	−35.5	−6.17	−29.32
hsa-miR-221	68	8:0:0	−25.6	−7.11	−18.48
hsa-miR-222	68	8:1:0	−24	−7.11	−16.88
hsa-miR-760	61	8:1:0	−20.99	−8.41	−12.57
hsa-miR-361-5p	42	8:1:0	−15.2	−3.4	−11.79
hsa-miR-103	68	8:1:1	−16.1	−7.11	−8.98
hsa-miR-107	68	8:1:1	−16.1	−7.11	−8.98
hsa-miR-1304	21	8:1:1	−11.8	−2.82	−8.97
hsa-miR-330-5p	59	8:1:0	−16.7	−8.64	−8.05
hsa-miR-612	57	8:1:1	−15.4	−7.42	−7.97
hsa-miR-1322	21	8:1:1	−10.5	−2.82	−7.67
hsa-miR-23a	67	8:1:1	−14.8	−7.16	−7.63
hsa-miR-23b	67	8:1:1	−14.8	−7.16	−7.63
hsa-miR-151-3p	39	8:0:1	−11.9	−4.36	−7.53
hsa-miR-1262	56	8:1:1	−13.7	−6.47	−7.22
hsa-miR-196b	55	8:1:1	−13.6	−6.46	−7.13
hsa-miR-1267	32	8:1:0	−10	−3.5	−6.49
hsa-miR-1290	27	8:1:1	−8.5	−3.03	−5.46

1 miRNA-binding position :MBS described in Figure 1 was analyzed in online microRNA-target prediction tool (http://genie.weizmann.ac.il/pubs/mir07/mir07_prediction.html)

2 Seed sequence matching to the MBS

3 Gibbs free energy for miRNA-MBS duplex

4 Gibbs free energy for unbound MBS

5 Gibbs free energy difference (=dGduplex-dGopen)

Table 1 and 2: Prediction of miRNA-sponge interaction on perfectly matched (Table 1) or bulged (Table 2) sponge unit (MBS). The sequence described in Figure 1 was analyzed in online microRNA-target prediction tool (Pictar, http://pictar.mdc-berlin.de) and top 20-ranked miRNA candidates are shown.

**Table 2:** List of miRNAs predicted to bind to the bulged miRNA sponge (MBS)

microRNA	Position^1^	Seed^2^	dGduplex^3^	dGopen^4^	ddG^5^
hsa-miR-21	40	8:1:0	−22	−5.02	−16.97
hsa-miR-221	61	8:1:0	−22	−8.49	−13.5
hsa-miR-222	61	8:1:0	−21	−8.49	−12.5
hsa-miR-155	15	8:0:0	−16.3	−4.07	−12.22
hsa-miR-196b	49	8:1:1	−19	−9.03	−9.96
hsa-miR-485–5p	55	8:1:0	−18.29	−9.19	−9.09
hsa-miR-1304	16	8:1:1	−12.7	−4.08	−8.61
hsa-miR-138	39	8:1:1	−13.6	−5.04	−8.55
hsa-miR-138	31	8:1:1	−13.87	−5.67	−8.19
hsa-miR-532–5p	35	8:1:1	−9.9	−1.78	−8.11
hsa-miR-612	51	8:1:1	−16.7	−9.08	−7.61
hsa-miR-874	34	8:1:1	−11.74	−4.24	−7.49
hsa-miR-758	37	8:1:1	−11.3	−4.19	−7.1
hsa-miR-645	56	8:1:0	−15.7	−9.19	−6.5
hsa-miR-448	59	8:1:1	−14.5	−8.45	−6.04
hsa-miR-567	37	8:1:1	−10	−4.19	−5.8
hsa-miR-602	58	8:1:1	−14.02	−8.32	−5.69
hsa-miR-1267	27	8:1:0	−9.2	−3.65	−5.54
hsa-miR-590–5p	40	8:1:0	−10.4	−5.02	−5.37

1 miRNA-binding position :MBS described in Figure 1 was analyzed in online microRNA-target prediction tool (http://genie.weizmann.ac.il/pubs/mir07/mir07_prediction.html)

2 Seed sequence matching to the MBS

3 Gibbs free energy for miRNA-MBS duplex

4 Gibbs free energy for unbound MBS

5 Gibbs free energy difference (=dGduplex-dGopen)

Table 1 and 2: Prediction of miRNA-sponge interaction on perfectly matched (Table 1) or bulged (Table 2) sponge unit (MBS). The sequence described in Figure 1 was analyzed in online microRNA-target prediction tool (Pictar, http://pictar.mdc-berlin.de) and top 20-ranked miRNA candidates are shown.

### The multi-potent miRNA sponge sensitizes cells to doxorubicin better than single miRNA sponge and increases apoptosis

**Figure 5:**
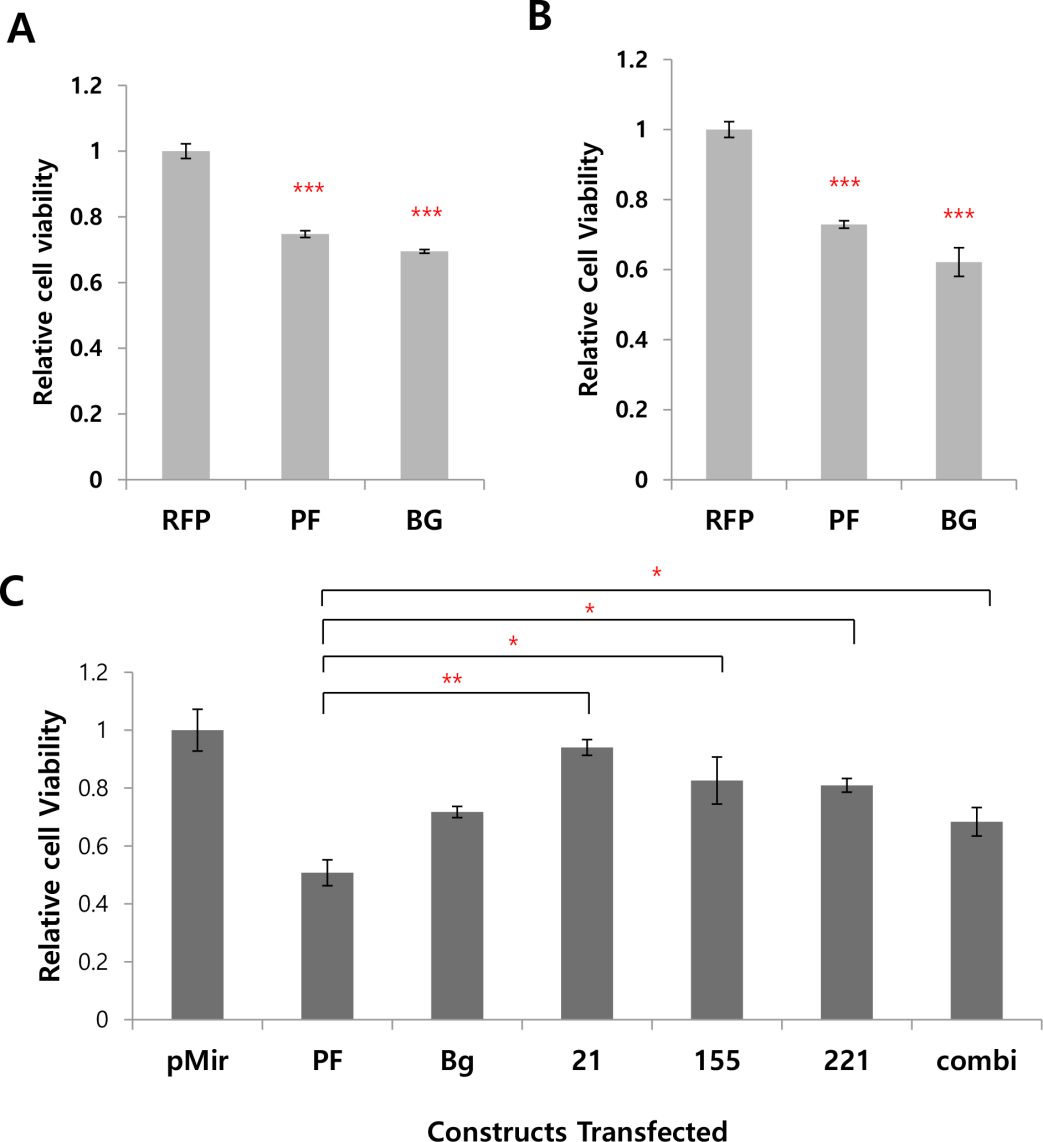

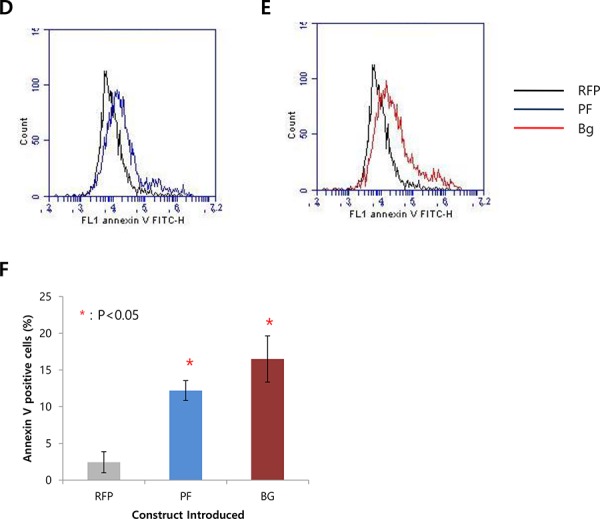
The expression of multi-potent miRNA sponge exerts antitumor activity **A, B.** Effect of the miRNA sponge to cancer drug (doxorubicin) sensitivity. In A, Flp-In T-REx 293 cells were treated with doxycycline (sponge induction) and doxorubicin together. In B, sponge was induced in cells and after 24 hours, doxorubicin was treated for another 24 hrs. The cell viability was measured by AlamarBlue assay. **C.** Comparison of the multi-potent miRNA sponge with single miRNA sponge for the anti-proliferative function. MDA-MB-436 cells were transfected with each of the construct indicated and after 48 hrs the cell proliferation was measured by AlamarBlue assay. (pMIR :pmiR-Report vector only; 21, 155, 222 : miR-21, miR-155, miR-222 targeting sponge respectively. Combi : mixture of the 3 kinds of single sponge (miRNA 21, 155, 221/222)). **D, E.** Effect of the miRNA sponge to apoptosis analyzed by Annexin V-staining, after doxorubicin treatment (20 hrs). FACS analysis shows more Annexin V-positive cells after sponge expression (RFP: control without MBS, marked as black line; PF: perfect sponge marked as blue line in D; Bg: Bulged sponge marked as red line in E. **F.** Quantification of the results from D and E. *p < 0.05, **p < 0.01, ***p < 0.001

Because we inhibited four oncogenic miRNAs by the sponge, we next questioned whether it ultimately affects the oncogenecity of cancer cells. To examine this, we treated doxycycline and doxorubicin to the inducible miRNA sponge cell line. After 24 hrs and 48 hrs after the doxorubicin treatment, cell proliferation was measured by AlamarBlue assay. As shown in Figure 5A and 5B, we could find significant reduction of the cellular viability in the miRNA sponge-induced cells. These data shows the inhibition of miRNAs can sensitize cancer cells to the drug. Based on this data, we then compared the antitumor activity of the multi-potent miRNA sponge with three other single miRNA-targeting sponges, where we introduced same copies of MBS but targeting for only one miRNA. As a result, we observed the multi-potent miRNA sponge inhibits cancer cell proliferation more than the single miRNA-targeting sponges (Figure 5C). The antitumor effect of the multi-potent miRNA sponge was further confirmed by measuring the apoptotic population from the drug treated cells with or without the induction of sponge (Figure 5D–5F). We observed significant increase of apoptotic cells after 20 hrs of doxorubicin treatment, for both of the perfect (D) and Bulged (E) sponge induced cells (quantified in F, with error bar). These data indicate the expression of miRNA sponge accelerates apoptosis induced by cancer drug.

### The expression of miRNA sponge inhibits cellular migration activity

**Figure 6:**
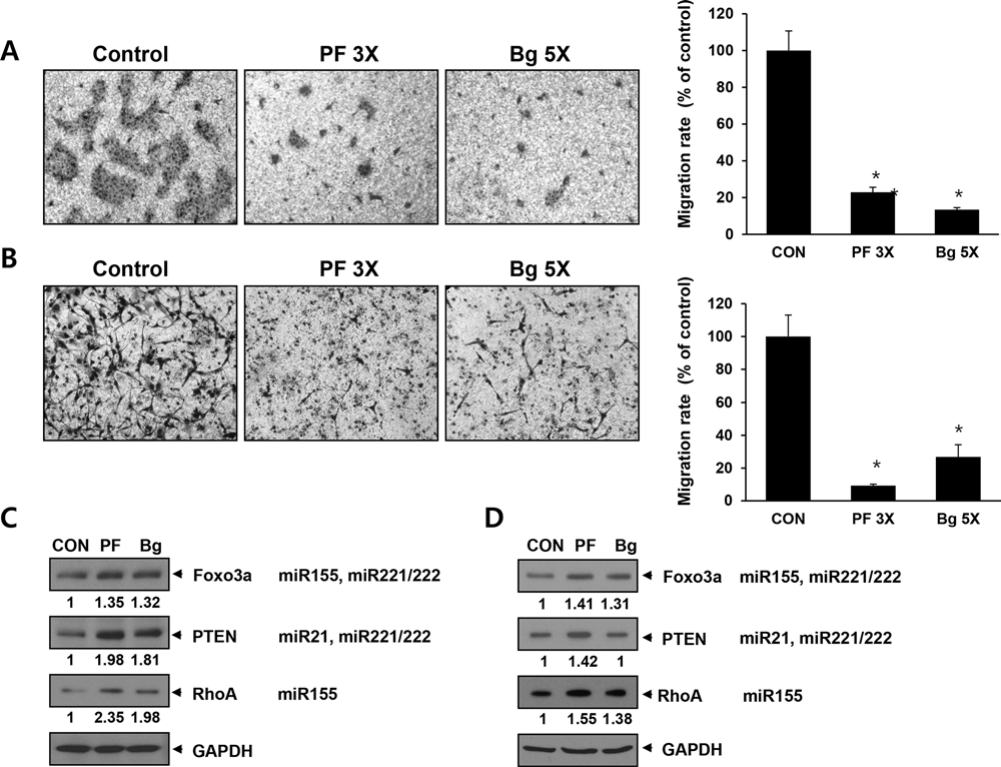
The expression of multi-potent miRNA sponge inhibits migration of cancer cells by up-regulating target proteins related to cell migration **A and B.** Migration assay in BxPC3 (A) and MDA-MB-436 (B) cells. Each picture is a representative image of the lower side of transwell. The migrated cells were photographed after 48 hrs of seeding onto transwell. Graphs on the right show cell number counted from the pictures on the left. **C** and **D.** Western blot analysis in BXPC3 (C) or MDA-MB-436 (D) for known miRNA targets related to cellular migration. The signal was measured by densitometry and normalized to GAPDH. *p < 0.05

Another important aspect of the oncogenic miRNA function is metastasis and the four miRNA we targeted are also involved in this process. Therefore, we tested if the miRNA sponge can inhibit cancer cell migration. We used transwell chamber to measure cell migration and the results are shown in Figure 6. Both of the BxPC3 pancreatic cells (A) and MDA-MB-436 (B) breast cancer cell showed dramatic decrease of the migration activity by the expression of either perfect (3X) or Bulged (5X) sponge. To gain a molecular insight for this change, we analyzed several target proteins involved in cell migration by western blotting and densitometry. The results in figure 6C (for BxPC3) and 6D (for MDA-MB-436) show 1.3∼2.3 fold of change in Foxo3a, PTEN and RhoA protein level. Previous reports demonstrated that increased level of the three proteins is associated with metastatic potential of the cancer cells [37–39]. These data demonstrates that the multi-potent miRNA sponge can sensitize cells to anticancer drug and inhibit migration, suggesting the simultaneous inhibition of multiple oncogenic miRNA can be a therapeutic intervention for cancer.

## DISCUSSION

There are a number of reports where miRNA sponge targeting single miRNA was successfully used [6–8]. Similarly, miRNA sponge has been generated to inhibit few unrelated miRNA sharing the same seed region [40]. Although some researchers tried to inhibit several miRNAs together, these miRNAs were generated from a polycistronic miRNA cluster [18, 33, 41]. In this study, we aimed to inhibit multiple (3∼4) oncogenic miRNAs simultaneously because in reality, there are more than one miRNA that might initiate or drive tumor progression [42]. To do this, the first thing to consider is how to choose these target miRNAs, as it is difficult to rank the importance of miRNA without knowing its exact targets and degree of inhibition. Additionally, each tumor can have different repertoire of driver miRNAs [43]. Therefore, we selected 4 miRNAs that are commonly demonstrated as up-regulated or oncogenic, based on the previous miRNA profiling and functional studies [26, 27, 31, 32, 34, 35].

Another important point needed to be considered for the construction of miRNA sponge was how to determine miRNA binding sequences (MBS) in the miRNA sponge, as recent reports suggested mismatch on the miRNA-inhibitor recognition can better recapitulate real miRNA-mRNA interaction so that show more efficient inhibition [40, 44]. To address this point, we introduced two types of MBSs, perfect and bulged (Figure 1). Interestingly, the capacity of miRNA sponge determined by the reduction of the luciferase activity showed a copy number dependent decrease of the activity for bulged sponge (Figure 2), whereas a single copy of MBS was enough to decrease the activity for perfect sponge. This difference might be due to the nature of miRNA-sponge interaction. In the bulged miRNA-sponge complex, the interaction will be less strong than the perfect one so that it needs more binding sites to increase the chance of capturing the target miRNAs.

As for the nonspecific binding of other miRNAs to the sponge, our real-time PCR analysis showed that is not remarkable for, at least, four top-ranked, non-specific miRNAs (Supplementary Figure 2). However, this results could not exclude a possibility that other miRNA still be able to bind to the sponge. Even if it happens, we think the binding will be much weaker than the four target miRNAs, so that impact will be negligible.

In order to evaluate the inhibitory effect of miRNA sponge, we tested a number of target proteins that are reported in literatures and listed as a target protein in MiRTarBase. We observed, however, some of the predicted target proteins are not increased and poor target level increase by the Bg sponge expression (Figure 4J). This result can be explained in several ways. First, the target inhibition can be cell line (i.e. context) dependent. Western blot analysis for the same proteins in MDA-MB-436 cells supports that the change of target protein could be dependent on the cell line (Supplementary Figure 4, compared to the results in Figure 4J). Second, there might be a range of miRNA inhibition that is sufficient to affect certain target protein expression. If it is true, then the critical factor, which determines the differential target regulation, would be the availability of the target (that is cell line dependent) and the affinity of the miRNA to the target (target sequence dependent). Based on this, we speculate some targets that were not altered by the Bg miRNA sponge could be the ones that have high affinity miRNA binding (so that it can compete with Bg (mismatched) sponge but not with PF sponge). If the level of such mRNA is relatively low, then the reduction of matching miRNA expression is not sufficient to abolish the repression of the protein level. Further study will be needed to prove this idea.

To investigate the functional output of the multiple miRNA inhibition, we examined cellular response to the cancer drug (doxorubicin) and migration after expression of the miRNA sponge. Because there are many miRNA targets involved in various kinds of cellular processes, the impact of the miRNA sponge can be explained in multiple ways. For example, Foxo3a is reported to suppress EMT in cancer cell whereas its down-regulation is known to promote metastasis [39]. On the other hand, Foxo3a is a negative regulator for Myc oncogene expression so the up-regulation of Foxo3a can inhibit cell proliferation [45]. Therefore, the up-regulation of Foxo3a by miRNA sponge results in multiple cellular responses. Likewise, up-regulation of PTEN can inhibit cellular migration via the suppression of AKT pathway that will also reduce cellular proliferation as well [46, 47]. We think that the antitumor function of our miRNA sponge can be shown in the other assays, given that the wide range of protein changes triggered by the inhibition of the target miRNAs.

Even though the exact mode of action for the multi-potent miRNA sponge is complex, we observed functional impact of the sponge is stronger than single miRNA targeting sponge (Figure 5C). We expect that the functional impact of the multi-potent miRNA sponge would be maximized not in a single type of functional assay (like proliferation or migration) but in an *in vivo* cancer model, where all of these miRNA-dependent cancer properties are reflected in survival rate.

In summary, the present study demonstrates a multi-potent miRNA sponge for a simultaneous inhibition of multiple oncogenic miRNAs, which showed antitumor function in cancer cells. More efforts in the development of delivery methods or the control of sponge expression in targeted tissue are needed for establishing the multi-potent miRNA sponge as an effective therapeutic candidate in the future.

## MATERIALS AND METHODS

### Cell culture and transfection

Human breast cancer cell lines MDA-MB-436, MCF-7 cell lines were cultured in RPMI media supplemented with 10% FBS and Penicillin-streptomycin (P/S). 293 Flp-In T-Rex cells were cultured with DMEM plus 10% FBS serum and P/S. Human pancreatic cancer cell lines Panc-1 and MIA-PaCa-2 were maintained in DMEM containing 10% FBS and 1% penicillin/streptomycin. BxPC3 cells were cultured in RPMI with 10% FBS. For transient sponge expression, cells were transfected using lipofectamine 2000 (Invitrogen) with the following sponge plasmids: EV (Empty vector; pMIR-report vector), Perfect 1X∼3X (pMIR-report vector with 1 to 3 copies of perfect-matched sponges at 3′UTR), Bulged 1X∼5X (pMIR-reporter vector with 1 to 5 copies of bulge-matched sponges at 3′UTR).

### Construction of miRNA sponge vector

We used pMIR-Report vector (Life technologies) as a backbone. Firstly, a linker consisting of Eco0109I and SanDI (for directional multimerization) was generated and inserted by SpeI/HindIII sites of the vector. The primer sequences for linker are as follows. Upper strand: 5′-Phos-CTAGT AGGGCCC GGGTCCC AGGGCCC A-3′; lower strand: 3′-A TCCCGGG CCCAGGG TCCCGGG TTCGA-Phos-5′. After that, an oligonucleotide pair harboring miRNA binding sites (MBS) of miR-155, miR21 and miR-221/222 with spacers (AATT) was designed and SanDI linker was added at the each end. This monomer unit was phosphorylated by PNK and ligated to the pMIR-Report / SanDI linker vector by SanDI site. This ligation generated monomerized as well as multimerized sponge vectors, which were controlled by changing a ratio between vector and insert. We generated multimerized sponge vector by increasing the molar ratio of vector: insert up to 1:100. The insert size was initially estimated by colony PCR with two primers annealed to right up/downstream of the SpeI/HindIII sites of the vector. The cloned sponge vector was sequenced to verify the correct MBS multimerization.

### Luciferase assay

Cells were seeded in 24-well plate and transfected with 150 ng of sponge or reporter plasmid DNA, which has one copy of miRNA binding site for four target miRNAs. As an internal control, 10 ng of SV40-Luc (Renilla luciferase) per well was used. Forty-eight hours after transfection, cells were lysed and luciferase activity was measured using Dual-Luciferase Reporter Assay System (Promega) and GloMax Luminometer (Promega), according to the manufacturer's protocol.

### Generation of inducible sponge and cell line

To generate inducible sponge, we introduced Flp-in pcDNA5/FRT-RFP vector that has RFP cloned into HindIII-XhoI sites (Generous gift from prof. SW Kang). The perfect and bulged sponge units, which were used in the luciferase-based sponges were inserted into the vector by ApaI site. This construct was transfected into Flp-In T-REx-293 Cell and selected with hygromycin (250 ug/ml) for 7 days. The resulting cells were tested for sponge induction after 24 hrs of doxycycline treatment (1 ug/ml), by checking the expression of RFP under fluorescence microscopy and western blotting. After sponge was induced by doxycycline, the expression of RFP was checked by fluorescence microscopy (Zeiss) and the pictures were taken at 200X magnification.

### Bioinformatics analysis for specificity prediction

We used a prediction tool from Segal Lab of computational biology (http://genie.weizmann.ac.il/pubs/mir07/mir07_prediction.html) as an algorithm to predict miRNA bindings for the sponge sequences generated. According to the prediction results, we selected four miRNAs (other than target miRNAs) that showed highest affinity to the sponge sequence for specificity test.

### Western blotting

Preparation of cell lysates and Western blot analysis were performed as previously described [48]. Membranes were probed with anti-RFP (Generous gift from Dr. Sang Wook Kang), Bim (1:1000, CST, Danvers, MA, USA), Stat5 (1:1000, CST, Danvers, MA, USA), Smad4 (1:1000, CST, Danvers, MA, USA), SRC3 (1:1000, CST, Danvers, MA, USA), Cyr61 (1:500, Santa Cruz Biotechnology, CA, USA), ATF2 (1:500, Santa Cruz Biotechnology, CA, USA), c-Jun (1:500, Santa Cruz Biotechnology, CA, USA), Bcl-2 (1:1000, CST, Danvers, MA, USA), and ER-alpha (1:500, Santa Cruz Biotechnology, CA, USA). As loading control, anti-beta-Actin (1:1, 000; Santa Cruz Biotechnology) antibody was used.

### miRNA real-time PCR analysis

For each sample, RNA extraction was performed using TRIzol (Invitrogen, Carlsbad, CA) following the instructions of the manufacturer. 1 ug of total RNA was used for cDNA synthesis (Superscript First-Strand Synthesis System; Invitrogen) following the manufacturer's protocol. For quantitative assessment of miR-155, TRIzol-isolated RNAs were reverse transcribed by miScript ll RT Kit and measured by miScript SYBR Green PCR Kit in LightCycler 480 II. Cycle threshold (Ct) values of the analyzed miRNAs were normalized to Ct values obtained for the noncoding, small nuclear RNA molecule U6. Data were expressed as fold change versus control.

### RNA Immunoprecipitation

Flp-In TREx 293 cells with inducible sponge (PF or Bg) were cultured in 100 mm dish till 90% confluence and doxycycline was treated for 24 hrs. Cells were washed twice with PBS and incubated with 2 ml RNA IP Lysis buffer (20 mM Tris-HCl at 7.5, 100 mM KCl, 5 mM MgCl_2_, 0.5% NP-40, protease inhibitors (Roche), RNase inhibitor (Fermentas), 10 mM DTT) for 10 min on ice. Anti-pan Ago clone 2A8 (MILLIPORE) and normal mouse IgG (oncogene) were used for RNA immunoprecipitation. Co-immunoprecipitated RNA was purified using RNAiso plus (TaKaRa) and the amount of sponge RNA was measured by real-time PCR using prime script™ RT Reagent Kit (TaKaRa) and Ampigene™ qPCR Green mix Hi-Rox (Enzo life sciences), in Light Cycler 480 II (Roche).

### Drug sensitivity assay

Cells were seeded in a 96 well plate at a density of 15, 000 cells/well in 100 ul culture media. To express the sponge, cells were treated with 1 ug/ml doxycycline after 12 hrs of seeding. 1 uM Doxorubicin was added to all cells 12 hrs after doxycycline treatment. To monitor cell proliferation, 1/10 volume of AlamarBlue^®^ reagent (Invitrogen) was added directly to control and drug treated cells after 36 hrs of the doxorubicin treatment. The cells were incubated for an additional four hours to measure viability, which was detected in a microplate fluorescence spectro-photometer (GenTek) by a protocol from the manufacturer. For the comparison of anti-proliferative activity of multi-potent miRNA sponge with single miRNA targeting sponges, MDA-MB-436 cells were seeded in 24-well plate at a density of 40000 cells/well in 500 ul culture media. After 24 hrs, cells were transfected with 200 ng of pMIR empty vector, perfect 2x, bulged 2x or single miRNA sponge, which has binding site (2X) for only one kind of target mRNA respectively. The transfected cells were analyzed by the same way described above.

### Annexin-V staining and FACS analysis

Flp-In T-REx293 cells were seeded in 6 well plates at 50–60% confluence. After 24 hrs, doxorubicin (1 uM) and doxycycline (1 ug/ml) were added to each well. After 20 hours, cells were harvested and stained according to the protocol of FITC-Annexin V Apoptosis Detection kit (BD Pharmingen). Briefly, trypsinized cells were washed twice with cold PBS and then resuspended in 1X binding buffer at a concentration of 1 × 10^6^ cells/ml. 100 ul of the solution was transferred to 1.5 ml tube. 5 ul of FITC Annexin V and 5 ul PI were added to each tube and incubated for 15 mins at RT in the dark. Then 400 ul of 1X binding buffer was added to each tube. Stained cells were analyzed by using an Accuri Flow Cytometry (BD Biosciences). The percentage of FITC-positive cells was calculated using the CFlow software.

### Migration assay

Migration ability was assayed by the use of Transwell chambers (Corning Costar) with 6.5-mm diameter polycarbonate filters (8-μm pore size). In brief, the cells were trypsinized and resuspended at a final concentration of 10^6^ cells/mL, in serum free medium. One hundred microliters of the cells' suspension were loaded into each of the upper wells. 10% FBS were used as chemo-attractants in the lower chambers, and the chamber was incubated at 37°C for 30 hours (BxPC3) or 48 hours (MDA-MB-436). The cells were fixed and stained with hematoxylin and eosin. Non-migrated cells on the upper surface of the filter were removed by wiping them with a cotton swab, and migration ability was measured by counting cells that had migrated to the lower side of the filter, by using an optical microscope (X100).

## SUPPLEMENTARY FIGURES AND TABLES


