# Supplementary material for: Simultaneous inhibition of multiple oncogenic miRNAs by a multi-potent microRNA sponge
Source: Oncotarget. 2015 Jul 30;6(24):20370–87. doi: 10.18632/oncotarget.4827 (PMC4653011; doi:10.18632/oncotarget.4827)
Supplement: Supplementary file 1 [file oncotarget-06-20370-s001.pdf]

SUPPLEMENTARY FIGURES AND TABLES

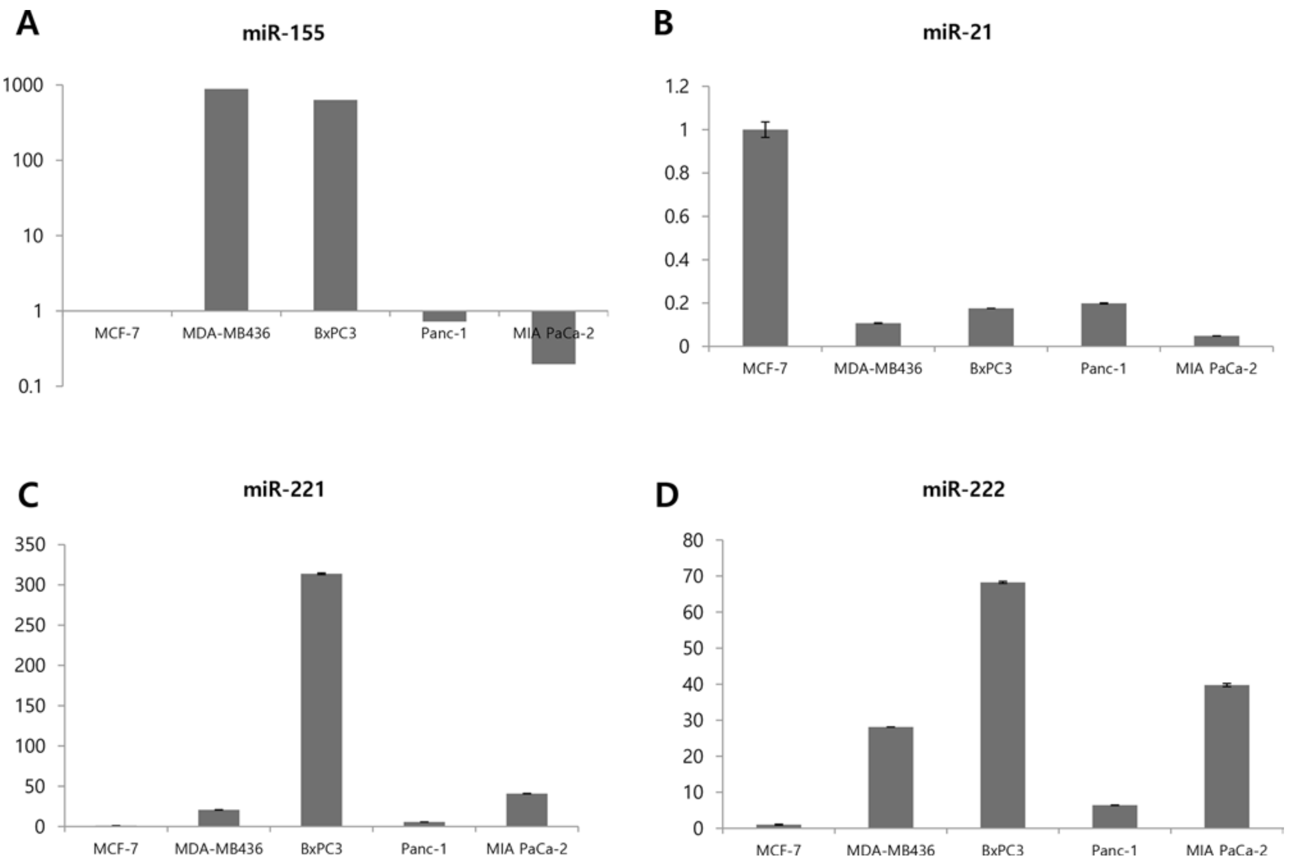

Supplementary Figure S1: Endogenous level of the four targeted miRNAs in breast and pancreatic cancer cells. The level of each miRNA was measured by real time PCR and indicated as relative value to the level in MCF7 cell. **A.** miR-155, **B.** miR-21, **C.** miR-222, **D.** miR-221

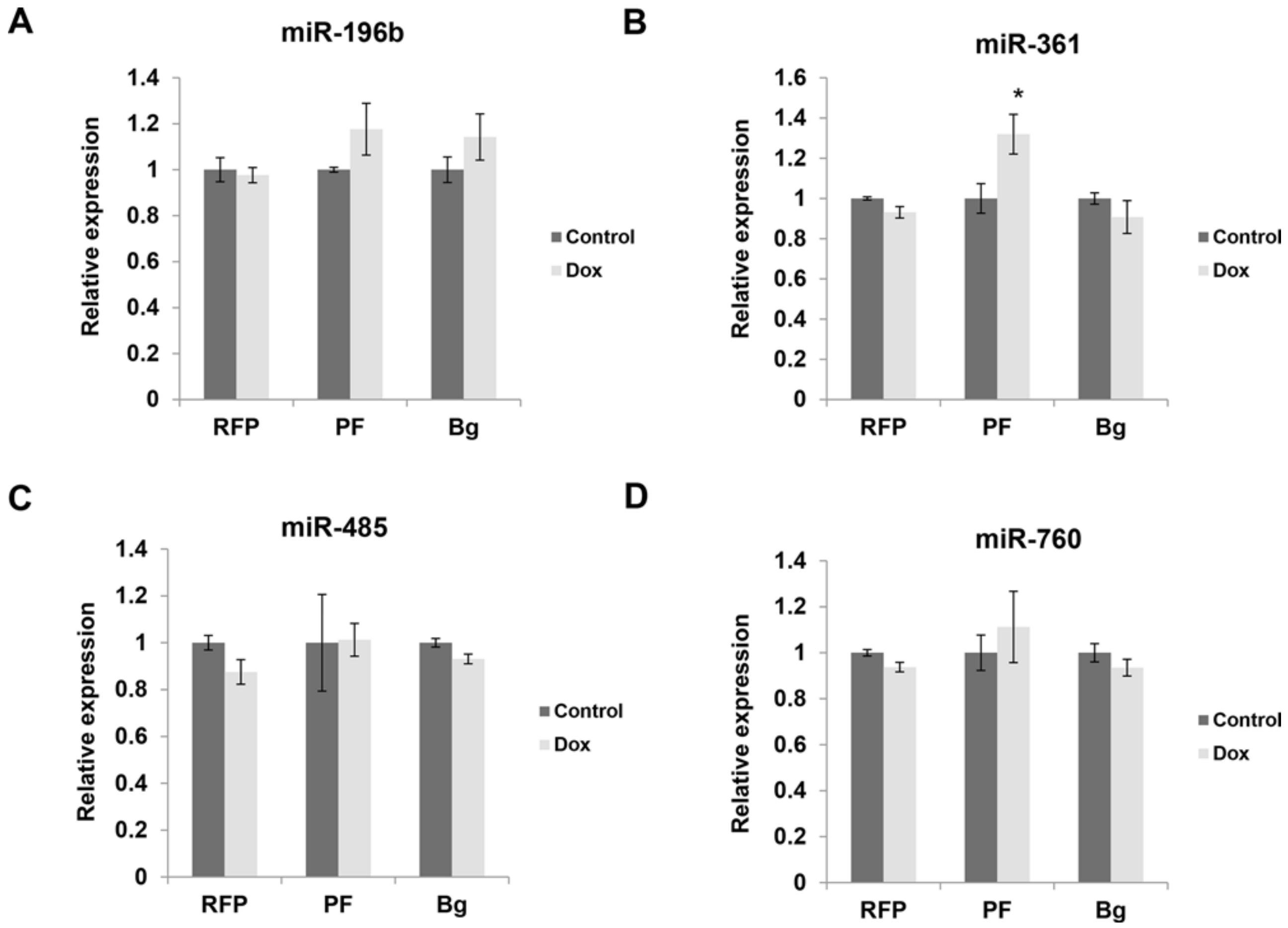

Supplementary Figure S2: Marginal non-specific inhibition by the multi-potent miRNA sponge. Real time-PCR quantification of the four miRNAs, which were predicted to have relatively high-affinity to the miRNA sponge. Note that marginal expression change of the four non-specific miRNAs by the expression of the miRNA sponge (PF: Perfect matched, Bg: Bulged).

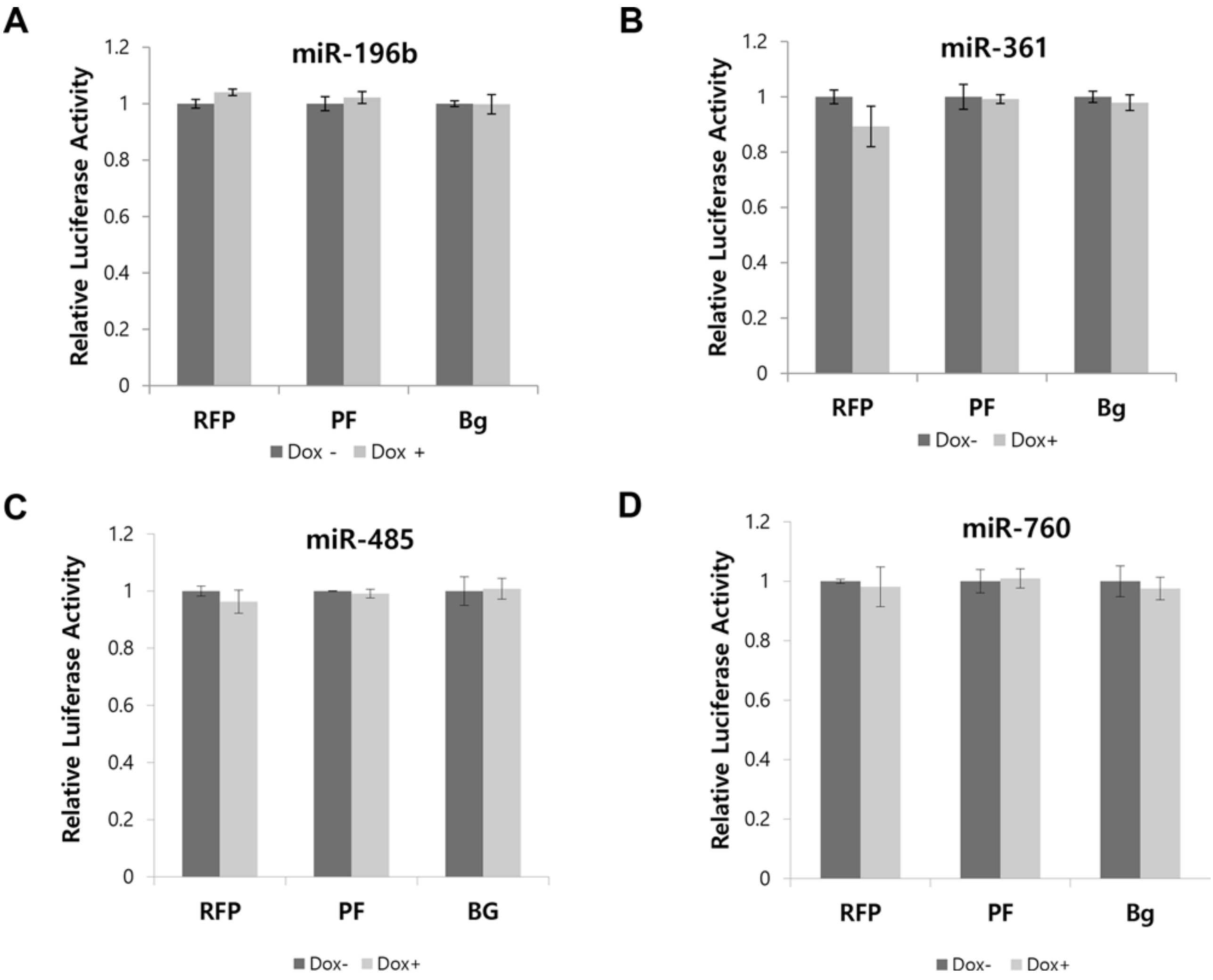

Supplementary Figure S3: Luciferase reporter assay for each miRNA, which were predicted to have relatively high-affinity to the miRNA sponge. (PF: Perfect matched, Bg: Bulged, Dox-: no doxycycline treatment, Dox+: doxycycline treatment).

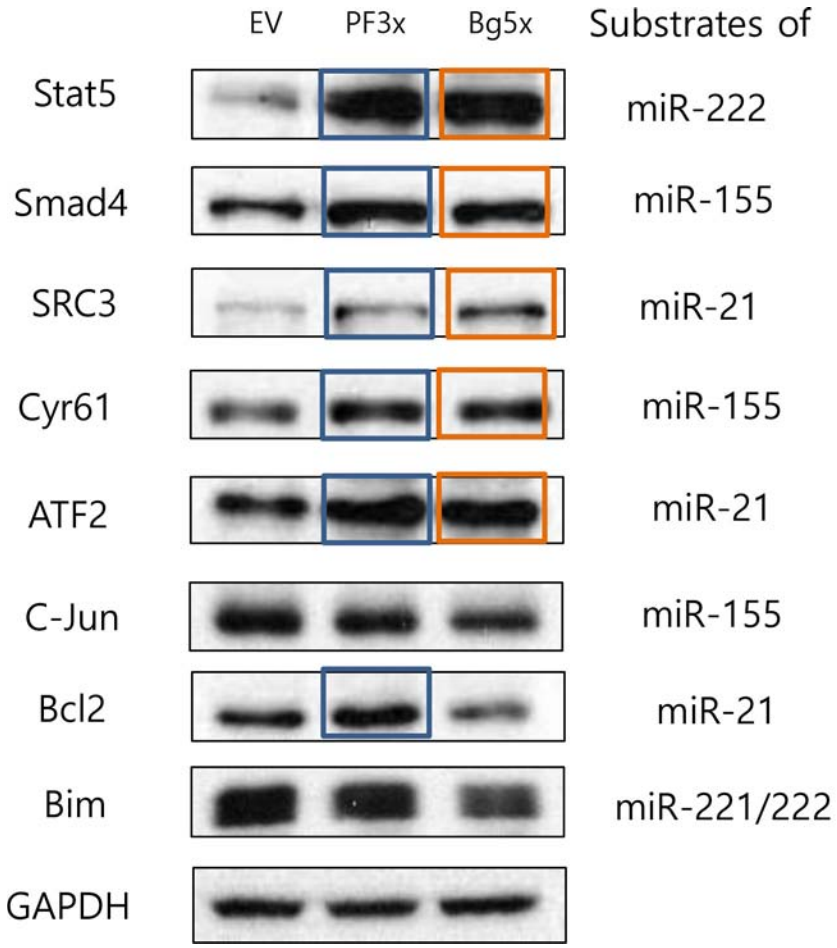

Supplementary Figure S4: Western blotting analysis of target proteins, which were shown in figure 4J. The protein levels were analyzed in MDA-MB-436 cell after transfection of perfect 3x or bulged 5x sponge DNA plasmids. Boxes indicate targets that showed increased protein level after expression of sponge.

Supplementary Table S1. Primer sequences used in this study

Primer Name	Sequences
1-Perfect_miR155_21_221222-SP_S	5′-GTC CCA CCC CTA TCA CGA TTA GCA TTA AAA TTT CAA CAT CAG TCT GAT AAG CTA AAT TAC CCA GAG CAA TGT AGC TGG-3′
2-Perfect_miR155_21_221222-SP_AS-1	5′-GAC CCA GCT ACA TTG CTC TGG GTA ATT TAG CTT ATC AGA CTG ATG TTG AAA TTT TAA TGC TAA TCG TGA TAG GGG TGG-3′
3-Bulged_miR155_21_221222-SP_S	5′-GTC CCA TTT TGT TTT AGC ATT AAA ATT TCA ACA TCA GGA CAT AAG CTA AAT TAC CCA GCC TAT GTA GCT GG-3′
4-Bulged_miR155_21_221222-SP_AS-1	5′-GAC CCA GCT ACA TAG GCT GGG TAA TTT AGC TTA TGT CCT GAT GTT GAA ATT TTA ATG CTA AAA CAA AAT GG-3′
5-Linker_SpeI_EcoO109I_SanDI_ EcoO109I_HindIII_S	5′-CTA GTA GGG CCC GGG TCC CAG GGC CCA-3′
6-Linker_SpeI_EcoO109I_ SanDIEcoO109IHindIII_AS-1	5′-AGC TTG GGC CCT GGG ACC CGG GCC CTA-3′
miR-155_rep_SanDI_For	5′-GTC CCA CCC CTA TCA CGA TTA GCA TTA AGG-3′
miR-155_rep_SanDI_Rev	5′-GAC CCT TAA TGC TAA TCG TGA TAG GGG TGG-3′
miR-21_rep_SanDI_For	5′-GTC CCT CAA CAT CAG TCT GAT AAG CTA GG-3′
miR-21_rep_SanDI_Rev	5′-GAC CCT AGC TTA TCA GAC TGA TGT TGA GG-3′
miR-221_rep_SanDI_For	5′-GTC CCG AAA CCC AGC AGA CAA TGT AGC TGG-3′
miR-221_rep_SanDI_Rev	5′-GAC CCA GCT ACA TTG TCT GCT GGG TTT CGG-3′
miR-222_rep_SanDI_For	5′-GTC CCA CCC AGT AGC CAG ATG TAG CTG G-3′
miR-222_rep_SanDI_Rev	5′-GAC CCA GCT ACA TCT GGC TAC TGG GTG G-3′
miR-196_SpeI_For	5′-CTA GTT AGG TAG TTT CCT GTT GTT GGG A-3′
miR-196_HindIII_Rev	5′-AGC TTC CCA ACA ACA GGA AAC TAC CTA A-3′
miR-361_SpeI_For	5′-CTA GTT TAT CAG AAT CTC CAG GGG TAC A-3′
miR-361_HindIII_Rev	5′-AGC TTG TAC CCC TGG AGA TTC TGA TAA A-3′
miR-485_SpeI_For	5′-CTA GTA GAG GCT GGC CGT GAT GAA TTC A-3′
miR-485_HindIII_Rev	5′-AGC TTG AAT TCA TCA CGG CCA GCC TCT A-3′
miR-760_SpeI_For	5′-CTA GTC GGC TCT GGG TCT GTG GGG AA-3′
miR-760_HindIII_Rev	5′-AGC TTT CCC CAC AGA CCC AGA GCC GA-3′
miR-222_SpeI_For	5′-CTA GTC TCA GTA GCC AGT GTA GAT CCT A-3′
miR-222_HindIII_Rev	5′-AGC TTA GGA TCT ACA CTG GCT ACT GAG A-3′
Ago2 RNA IP real time PCR primer	5′-TTG GTT ACG GGG CCC CTG GG-3′
	5′-GCG CCT AGA GGG CCC GGG TC-3′

Supplementary Table S2. miRNA sequences targeted in this study obtained from miRBase

miRNA	miRBase ID	sequence(s)
miR-155	MIMAT0000646	uuaaugcuaaucgugauaggggu
miR-21	MIMAT0000076	uagcuuaucagacugauguuga
miR-221	MIMAT0000278	agcuacauugucugcuggguuuc
miR-222	MIMAT0000279	agcuacaucuggcuacugggu

(www.mirbase.org)
